# Automatic Depression Detection Using Smartphone-Based Text-Dependent Speech Signals: Deep Convolutional Neural Network Approach

**DOI:** 10.2196/34474

**Published:** 2023-01-25

**Authors:** Ah Young Kim, Eun Hye Jang, Seung-Hwan Lee, Kwang-Yeon Choi, Jeon Gue Park, Hyun-Chool Shin

**Affiliations:** 1 Medical Information Research Section Electronics and Telecommunications Research Institute Dajeon Republic of Korea; 2 Clinical Emotion and Cognition Research Laboratory, Inje University Goyang Republic of Korea; 3 Department of Psychiatry, Inje University, Ilsan-Paik Hospital Goyang Republic of Korea; 4 Bwave Inc Goyang Republic of Korea; 5 Department of Psychiatry, College of Medicine, Chungnam National University Daejeon Republic of Korea; 6 Artificial Intelligence Research Laboratory Electronics and Telecommunications Research Institute Dajeon Republic of Korea; 7 Tutorus Labs Inc Seoul Republic of Korea; 8 Department of Electronics Engineering, Soongsil University Seoul Republic of Korea

**Keywords:** depression, major depressive disorder, MDD, automatic depression detection, ADD, mobile health, deep learning, speech analysis, acoustic, mobile phone, smartphone

## Abstract

**Background:**

Automatic diagnosis of depression based on speech can complement mental health treatment methods in the future. Previous studies have reported that acoustic properties can be used to identify depression. However, few studies have attempted a large-scale differential diagnosis of patients with depressive disorders using acoustic characteristics of non-English speakers.

**Objective:**

This study proposes a framework for automatic depression detection using large-scale acoustic characteristics based on the Korean language.

**Methods:**

We recruited 153 patients who met the criteria for major depressive disorder and 165 healthy controls without current or past mental illness. Participants' voices were recorded on a smartphone while performing the task of reading predefined text-based sentences. Three approaches were evaluated and compared to detect depression using data sets with text-dependent read speech tasks: conventional machine learning models based on acoustic features, a proposed model that trains and classifies log-Mel spectrograms by applying a deep convolutional neural network (CNN) with a relatively small number of parameters, and models that train and classify log-Mel spectrograms by applying well-known pretrained networks.

**Results:**

The acoustic characteristics of the predefined text-based sentence reading automatically detected depression using the proposed CNN model. The highest accuracy achieved with the proposed CNN on the speech data was 78.14%. Our results show that the deep-learned acoustic characteristics lead to better performance than those obtained using the conventional approach and pretrained models.

**Conclusions:**

Checking the mood of patients with major depressive disorder and detecting the consistency of objective descriptions are very important research topics. This study suggests that the analysis of speech data recorded while reading text-dependent sentences could help predict depression status automatically by capturing the characteristics of depression. Our method is smartphone based, is easily accessible, and can contribute to the automatic identification of depressive states.

## Introduction

Depression is a serious psychiatric illness affecting >300 million people worldwide. It leads to a variety of negative health outcomes in individuals [1,2]. When left untreated, it can affect the quality of life, lower work productivity, and lead to suicide [3]. When diagnosed correctly, depression is a treatable disorder, and its symptoms can be relieved [4]; however, an accurate diagnosis of major depression is difficult because it is a biologically and clinically heterogeneous entity [5]. In addition, it can be difficult to access trained clinicians in a timely manner, and the diagnosis process and quality are inconsistent for patients in need of professional assistance [6]. There is an urgent need to develop a method for reliable automatic diagnosis and timely screening of depression to facilitate remote assessments and more precise treatment with personalization [7].

A promising approach to address the abovementioned problems is to identify depression markers and advanced machine learning (ML) techniques using real-world accessible sensors (eg, wearables, cameras, and phones). These approaches may make it easier for nonspecialists to effectively identify symptoms in patients with depression and accordingly direct them toward appropriate treatment or management. Previous studies have explored a spectrum of behavioral signal approaches, such as speech [7,8], text [9-11], facial expressions [12-14], and body movements [15-18], to develop depression assessment. Among these, speech has proven to be a reliable biomarker for depression assessment [19,20] and is popular because of its accessibility and availability compared with other behavioral signals, making it ideal data for depression screening. Moreover, it requires significantly less bandwidth and lower processing power, thus making it a simple and computationally inexpensive implementation of depression detection.

Automatic depression detection (ADD) has gained popularity with the advent of publicly available data sets [21] and the power of ML techniques to learn complex patterns. Among speech-based methods, previous studies have focused more on using handcrafted acoustic features, such as prosody [13], formant [22], and cepstral [23] features, and then classifying patterns using ML algorithms, such as support vector machine (SVM) [24], logistic regression [25], and random forest (RF) [26]. These studies have suggested that acoustic features are closely related to depression. However, handcrafted acoustic features require considerable effort and time, and because the extracted features depend on the researcher’s domain knowledge, some useful information related to depression may be lost. Demonstrating the reliable acoustic features of ADD remains an open research challenge. More recently, deep learning techniques have achieved high success in audio and video recognition tasks, and many studies have reported that deep learning approaches in depression detection have significantly improved performance compared with conventional approaches that use partial representation [27]. These approaches have greatly improved performance because they can automatically learn effective hierarchical representation of speech without human intervention [28]. Therefore, in this study, we explore how depression detection can benefit from deep learning.

Text-dependent read speech helps to reduce acoustic variability, as reading language content can be designed to express behavior in a controlled manner, such as the same length and content [29]. Therefore, using it for depression detection can provide a more precise performance even under limited conditions. Stasak et al [30] observed that, unlike the spontaneous mode, text-dependent affective read speech provides a more accurate ground truth for speech analysis because of speech and affective constraints. They argued that it has the advantage of being used stably for automatic depressive detection in clinical settings. Speech analysis requires the standardization of speech acquisition to produce consistent results. Therefore, a focus on text-dependent read speech that includes every sound and represents normal speech can have uniformity in speech derivation procedures that can trigger the characteristics of voice [31]. However, previous studies on text-dependent read speech–based depression detection have not investigated pathological acoustic qualities in a more sizable depression data set. In addition, cohort studies in English dominate this field, and prospective data on improved access to psychiatric care among non-English speakers are lacking [32].

To address these issues, we used audio samples based on text-dependent reading modes to determine whether depressive symptoms could be detected based on differences in acoustic characteristics. We believe that an autosensing approach using deep learning to describe text-dependent reading modes can significantly contribute to this field of research. Therefore, in this study, a Korean-based speech cohort was used to diagnose depression using text-dependent read speech. We then applied a convolutional neural network (CNN) layer and a dense layer to model the depressive state. Finally, the trained model was used to predict depression among unknown data samples. The performance of the model was compared with that of other approaches, ML, and pretrained models. The results show that our approach is effective in easily and automatically assessing depressive states in speech.

## Methods

### Recruitment

The participants were 153 patients with major depressive disorder (MDD) and 165 healthy controls. All participants were from South Korea. The inclusion criterion was that study participants should be aged ≥19 years. All patients with MDD were evaluated by board-certified psychiatrists according to the Diagnostic and Statistical Manual of Mental Disorder criteria to identify their current mood states. The severity of depressive symptoms was assessed using the Hamilton Depression Rating Scale (HAM-D) and Patient Health Questionnaire-9 (PHQ-9) [33]. Patients with bipolar disorder, schizophrenia, other psychotic disorders, delirium, dementia, mental retardation, alcohol use disorders, organic mental disorders, neurological illnesses, brain damage, and serious medical illnesses were excluded. Healthy participants without any medical history of psychiatric disorders were recruited via advertisements to form the control group. The study procedures were fully explained to all participants, and they provided written consent for the use of data for the study objects before study enrollment. In addition, each participant was paid US $50.

### Ethics Approval

This study was reviewed and approved by the Institutional Review Boards of Inje University Ilsan Paik Hospital (number 2019-12-011-015) and Chungnam National University Hospital (number 2019-10-101-018), which specializes in mental health in South Korea.

### Reading Task-Based Experimental Design and Speech Data Sets Acquisition

**Figure 1:**
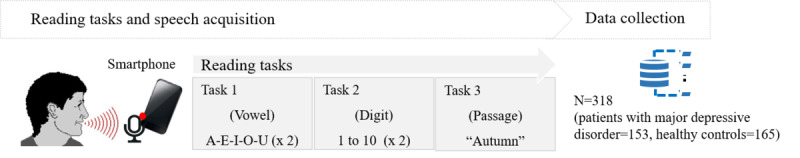
Experimental procedure based on the read-dependent speech tasks.

The experimental protocol in this study was designed to assess speech responses to identify depression using predefined text-dependent speech tasks (Figure 1). The protocol consisted of three automatic speech reading tasks: (1) vowel; (2) digits from 1 to 10; and (3) a passage in Korean called “Autumn” [34], a standard 118-word paragraph. The “Autumn” is a standard text used to evaluate phonetic utterance in Korea, which has a balanced proportion of consonants and vowels in Hangeul and does not have an intended sentiment. The vowel and digit tasks were repeated 2 times. For example, participants were instructed to read a word or sentence from scripts. The experimental guide presented the participants with standardized instructions and examples before recording the speech samples. The primary language of the participants was Korean.

The reading tasks of each participant were recorded using a smartphone (built-in microphone of Samsung Galaxy S10). Speech samples on the microphone were saved as mono-PCM (Pulse-Code Modulation; 32 bits) .wav files, sampled at 44.1 kHz. The speech samples were recorded in a quiet room, and the microphone was positioned at a distance of approximately 30 cm from the participants to ensure the quality of the recordings. We created slides with scripts for each task and placed them on a table where the participants could easily see them. The participants were instructed to read the script through the slides in a comfortable and self-selected pitch and volume. The collected audio data set was used for the experiments in this study.

### Preprocessing for Audio Signals Enhancement

The recorded audio signal included the speaker’s speech and nonspeech parts, such as silence and background noise caused by environmental factors. These nonspeech parts can be an obstacle to training acoustic features for depression recognition [35,36]. Therefore, the noise and silent parts were removed from the audio signals before extracting the acoustic features. In this study, the audio signal was removed using a Python package called *Noisereduce* [37], which implements stationary noise reduction based on the first 0.5 seconds of each utterance. The silent parts of the audio signal were removed using a voice activity detector [38] to obtain a record of the continuous speech parts. Low-quality recordings were excluded. After these processes, 318 audio samples were available, and the duration of the recordings for each task is described in Table 1.

**Table 1:** Time information of audio samples in the major depressive disorder (MDD) and healthy controls (HC) groups during the vowel, digit, and passage tasks.

			Vowel		Digit		Passage
**MDD**							
	Number of samples, n (%)	153 (48.1)		153 (48.1)		153 (48.1)	
	Speech time (seconds), mean (SD)	14.67 (4.07)		16.02 (5.39)		91.80 (17.46)	
	Total speech time (seconds)	2307		2373		14,150	
**HC**							
	Number of samples, n (%)	165 (51.9)		165 (51.9)		165 (51.9)	
	Speech time (seconds), mean (SD)	13.59 (4.06)		14.39 (4.44)		72.71 (7.91)	
	Total speech time (seconds)	2325		2361		13,110	

### Deep Learning Implementation for Speech-Based Depression Detection

**Figure 2:**
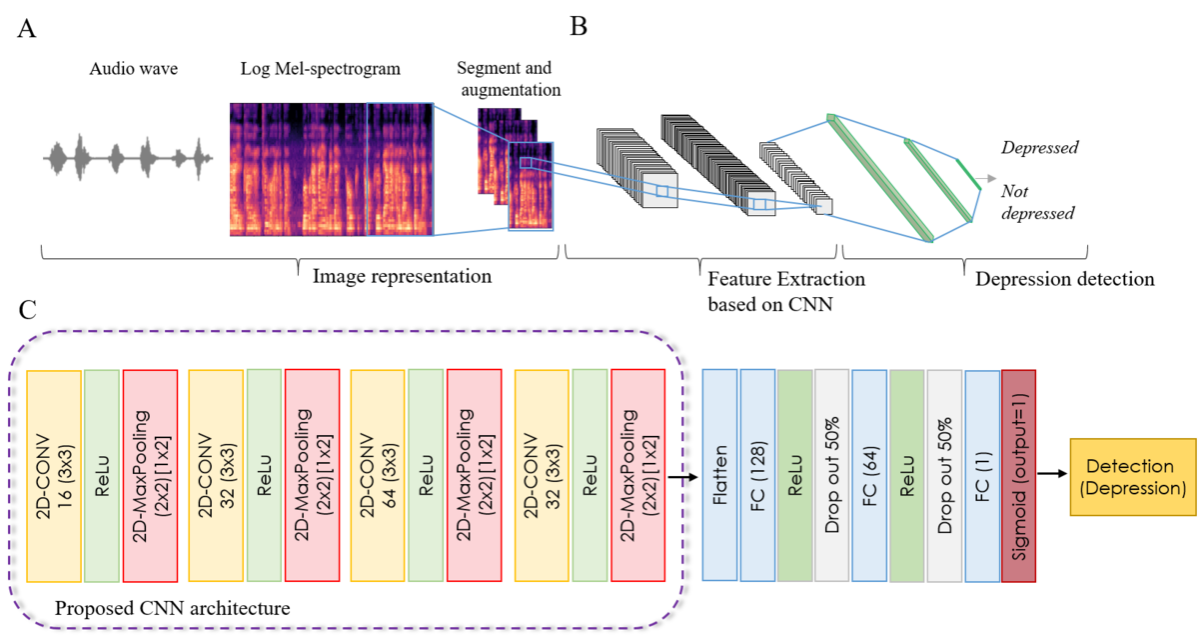
The pipeline of the proposed method for depression detection. (A) Image representation using spectrograms of audio samples, (B) Log-Mel spectrogram-based convolutional neural network (CNN) and depression detection, and (C) architectures on the proposed CNN model in this study. CONV: convolution; ReLu: rectified linear unit; FC: fully connected.

The goal of the proposed approach is to capture a series of acoustic features from audio samples using text-dependent speech tasks and map them into a similar representation space to determine the presence of depression. As CNN is a powerful framework for learning a feature hierarchy, it can provide a representation space capable of detecting depression. Thus, we chose the CNN architecture to learn spatially invariant features of audio samples. We trained a deep CNN from scratch and evaluated its classification performance. Figures 2A and 2B demonstrate the pipeline of the proposed method for depression detection. This method consists of 3 main processes: image representation using a log-Mel (LM) spectrogram, deep feature extraction using a deep CNN architecture, and a classifier. Next, we compared the discriminating ability of the CNN architecture with that generated by transfer learning using a well-known pretrained deep network. In addition, we confirmed whether the feature extraction approach using a deep CNN has advantages over the conventional ML model method in text-dependent speech tasks. Pretrained CNN model architectures and components are described in the *Pretrained CNN Models* section.

The audio signal must be properly converted to enable deep CNN input, and deep CNN training requires a large amount of labeled data to use deep feature extraction using a CNN for audio samples. To address this issue, we segmented the audio signal into an optimal length to enable depression recognition in each segment, as shown in Figure 2A. We adopted fixed-length frames with a duration of 25 ms and 50% overlap as the augmented samples [39,40]. In this study, each frame was transformed into 64 channels LM spectrogram with a size of 64×200 pixels as image-like patches. The parameters were as follows: n_ffs=1024 and hop_length=512. We then converted the spectrogram (mel_db) using a mel-scale. The LM spectrogram is a widely used method in audio applications using deep learning, which requires a 2D image representation of an audio signal with complex features [20,41,42]. After feature extraction, the audio samples were generated as a set of frame-level–based 2D LM spectrograms to conduct feature learning with a 2D CNN. Technically, these methods were implemented using the Librosa library.

We adapted augmentation techniques to increase the size of the training set to improve generalization. An augmentation method that generates new additional training data is a successful method for reducing model overfitting on sparse data and improving generalization performance. SpecAugment is the latest augmentation method developed by Google for spectrograms of input audio [43]. SpecAugment has shown a remarkable improvement in automatic speech recognition performance. This study focuses on 2 strategies of SpecAugment to modify the spectrogram by masking blocks of consecutive frequency channels and time channels. Frequency masking is applied over the f consecutive frequency channels in the range (f0, f0+f), where f is chosen from a uniform distribution (0, F) and f0 is chosen from (0, v-f), where F and v represent the maximum width of the frequency masks and the number of LM frequency channels, respectively. Similarly, time masking is applied over the t consecutive time steps in the range (t0, t0+t), where t is chosen from a uniform distribution (0, W) and t0 is sampled from (0, T-t), where W and T represent the maximum width of the time masks and the number of time steps, respectively. On the basis of the approach adopted by Park et al [43], spectrogram augmentation was applied through temporal masking, frequency masking, and mixed time and frequency masking. Including the raw training sample, this data augmentation procedure achieved a 4-times increase in the training data set.

We designed a CNN architecture to extract features from an LM spectrogram. The detailed model structure of the proposed CNN is shown in Figure 2C. Our CNN architecture consists of 4 blocks with a convolutional layer followed by a rectified linear unit as the activation function and 2×2 max-pooling with a 1×2 stride over the last 2 dimensions (time and frequency) for dimensionality reduction of the feature maps. A kernel of size 3×3 with 16, 32, 64, and 32 channels in 4 convolution blocks was used for the convolution operation. The feature set obtained from the last layers is required by the fully connected layers for classification.

### Pretrained CNN Models

In addition to the proposed CNN architecture, pretrained CNN models were used as feature extractors to evaluate depression detection in this study. We tested the following additional pretrained CNNs designed to extract audio representations: 4 state-of-the-art image CNN models, namely, VGG16, VGG19 [44], ResNet50 [45], Inception-v3 [46], and MobileNet-v2 [47], and 2 state-of-the-art sound event detection CNN models, namely, VGGish [48] and YAMNet [49].

The image CNN models Inception-v3, VGG16, VGG19, ResNet50, and MobileNet-v2 were also used with LM spectrogram images as the 2D input. The LM spectrograms were resized to 224×224 images with 3 channels (R, G, and B) because of their suitability for deep feature extraction. For the classification of the image CNN models, we applied global average pooling instead of using a flattening layer after the feature extractor. For all these models, the final fully connected layers were eliminated and redesigned in the new classifier.

VGGish is a deep audio embedding method for training a modified visual geometry group architecture to predict video tags from the Youtube-8M data set [50]. It extracts 128-dimensional embedding from 0.96 seconds of audio. YAMNet uses the MobileNet _v1 depth-wise separable convolution architecture and is trained on the AudioSet corpus [51]. It extracts 1024-dimensional embedding from 0.96 seconds of audio. The input sizes of VGGish and YAMNet are 94×64 for the LM spectrogram, and they transform audio input into a high-level dimensional embedding vector of 128 and 1024, respectively, as a classification input.

For the proposed CNN and pretraining models, the classifier uses 128 and 64 fully connected dense layers, respectively, and rectified linear unit activation. We use a 0.5 dropout probability between dense layers to prevent overfitting of the training data. The output layer consisted of 1 hidden unit to classify an image into 2 classes: MDD and control. For the output layer, we used a sigmoid activation function for binary classification.

The training process was set up to run 20 epochs for all the CNN models. For model training, the Adam optimizer was selected with a fixed learning rate of 1e-3. In this study, the learning rate was based on the validation accuracy of the model, and early stopping was performed. All the parameters of the testing stage were applied similar to the training stage. The deep CNN was implemented using Python (version 3.7.11) and TensorFlow (version 2.5.0) on a Quadro RTX 8000 48GB GPU (NVIDIA).

### Depression Detection Based on the Conventional ML Model

The baseline audio signals extracted 3 widely used acoustic feature representations—Mel-frequency cepstral coefficients (MFCCs), extended Geneva Minimalistic Acoustic Parameter Set (eGeMAPS) [52], and Interspeech Computational Paralinguistics Challenge (COMPARE) [53] features—to compare the effectiveness of the deep spectral representations model. The suitability of these acoustic representations for speech-based emotion recognition tasks has been demonstrated in previous studies [54,55]. MFCCs, which are widely used in the field of speech recognition, exhibit short-term power spectral features extracted from acoustic speech signals and reflect changes in the human vocal tract [56]. In a previous study [57,58], MFCC was demonstrated to be highly effective in detecting depression compared with other speech features in conventional approaches. We explored whether MFCCs as diagnostic biomarkers of depression could allow discrimination between patients with depression and control participants in this study. MFCCs were calculated from the window interval of the audio signal. We used MFCCs calculated by using a frame length of 25 ms with 50% overlap and a Hamming window. The frame of the audio data was transformed using a Fourier transform and a 26 channel mel-scale filter bank. MFCCs from 1 to 12 were calculated logarithmically and obtained using a discrete cosine transform. The mean and SD of the MFCCs within the utterance segments were calculated. We also extracted the eGeMAPS and COMPARE low-level feature sets using the openSMILE toolkit [59]. The 38 eGeMAPS feature sets and 65 COMPARE feature sets were adapted, consisting of low-level descriptors capturing spectral, cepstral, prosodic, and voicing-related dynamic information from the audio signal. All feature sets were fed into multiple ML models of SVM, linear discriminate analysis (LDA), k-nearest neighbor (kNN), and RF implemented using the scikit-learn toolbox [60]. For the SVM classifier, we used a linear kernel function with a regularization parameter C=1. For the kNN classifier, the Euclidean distance was used as the distance metric, and the kNN was set to 5 as a parametric search from 1 to 5. RF consisted of 100 trees, and the Gini diversity index was used.

### Evaluation Criteria

The 318 audio data sets were randomly split into 254 training sets, 32 validation sets, and 32 testing sets in a balanced ratio between patients with MDD and healthy controls during the experiments. Participant-independent splits were used for training, validation, and testing.

A 10-fold cross-validation method was used to split the data and evaluate the performance of each classifier. We compared the predicted results with test data to evaluate their performance. In our study, we adopted the accuracy, precision, recall, receiver operating characteristic curve, and *F*_1_-score, by using the confusion matrix, as the evaluation criteria. The area under receiver operating characteristic curve (AUC) was also calculated.

## Results

### Demographic and Clinical Characteristics of Participants

The demographic and clinical characteristics of the age, education, HAM-D, and PHQ-9 factors were not normally distributed; these factors were compared between the control and MDD groups using the Mann-Whitney *U* test. Sex, alcohol history, and smoking history were compared using the chi-square test.

The descriptive demographic and clinical characteristics of the participants in this are summarized in Table 2. We recruited 153 patients (102 females) diagnosed with MDD and 165 healthy controls (123 females). There were no significant differences in the sex ratio and age between these groups. Significant differences were observed in years of education (*P*<.001). In addition, the MDD group had a significantly higher HAM-D (*P*<.001) and PHQ-9 than the control group.

**Table 2:** Demographic characteristics of major depressive disorder (MDD) and health controls (HC) groups.

Factors		MDD	HC	*P* value	
**Gender, n (%)**					.16^a^
	Male	51 (33.3)	42 (25.5)		
	Female	102 (66.7)	123 (74.5)		
Age (years), mean (SD)		38.64 (13.26)	37.15 (12.18)	.06	
Education (years), mean (SD)		13.10 (2.57)	14.71 (2.19)	*<.001* ^b^	
Nonalcoholic, n (%)		55 (35.95)	83 (50.30)	*.008* ^a,c^	
Nonsmoker, n (%)		45 (29.41)	21 (12.73)	*.006* ^a,c^	
HAM-D^d^, mean (SD)		22.18 (6.34)	2.07 (4.61)	*<.001* ^b^	
PHQ-9^e^, mean (SD)		16.92 (6.99)	2.14 (3.44)	*<.001* ^b^	

^a^Chi-square test.

^b^*P* values in italics are significant (*P*<.001).

^c^*P* values in italics are significant (*P*<.01).

^d^HAM-D: Hamilton Depression Rating Scale.

^e^PHQ-9: Patient Health Questionnaire-9.

### Testing Results

In this section, we analyze the classification capability of the proposed approach, including the conventional ML models and the CNN method, to recognize depression in unknown audio samples (test set) obtained from each text-based speech task.

First, we investigated the ability of the conventional ML methods to classify the samples as MDD or non-MDD for each data set of the vowel, digit, and passage tasks. Tables 3-5 show that the classification performance of MFCC, eGeMAPS, and COMPARE feature sets extracted from each data set were compared with the accuracy, precision, recall, and *F*_1_-score from the SVM, LDA, kNN, and RF classifiers. The results in Table 3 show that the RF classifier achieved a high accuracy of 72.80%, 73.70%, 72.14%, and 72.04% accuracy, precision, recall, and *F*_1_-score, respectively, compared with other classifiers when applying the COMPARE feature set on the vowel data set. As shown in Table 4, the accuracy for classification performance of the digit task was as high as 73.44% for the RF with the COMPARE feature set. On the data set of the passage task, we achieved accuracies of 70.73%, 69.95%, 68.54%, and 68.75% for SVM, LDA, RF classifier with the MFCC features, and RF classifier with the COMPARE feature set, respectively (Table 5).

**Figure 3:**
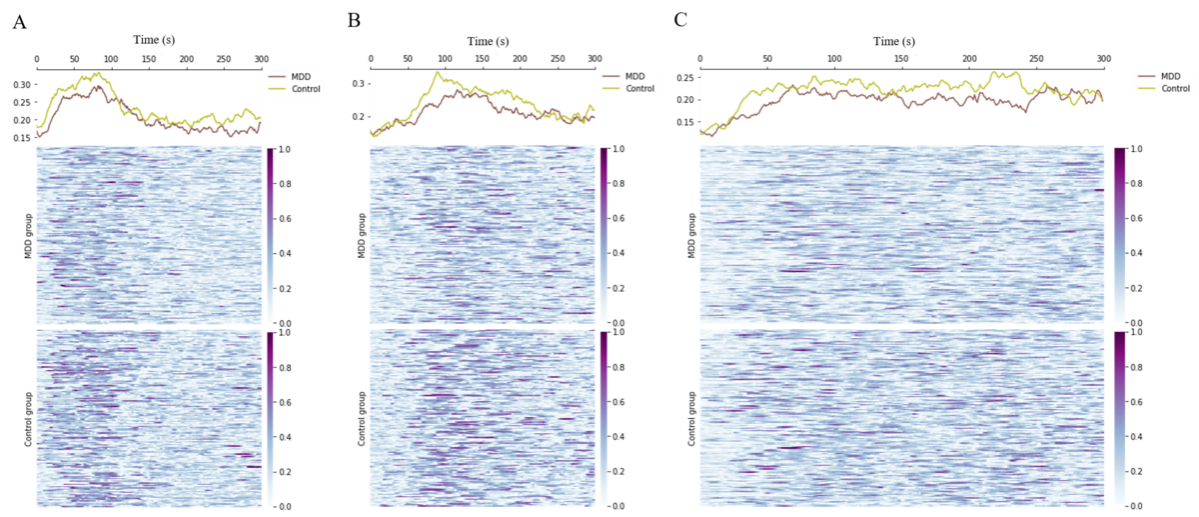
The Mel-frequency cepstral coefficient 33 variability between the major depressive disorder (MDD) and control groups in (A) vowel, (B) digits, and (C) passage tasks.

We further analyzed the variability of MFCCs between the 2 groups in each task to explore the differences in MFCCs between the MDD and control groups. As a result of analyzing the difference between the 2 groups among the 12 MFCCs, MFCC3 showed the greatest difference. Figure 3 shows the MFCC3 variability between the 153 MDD and 165 control groups and the sum of the variability of all participants. Here, variability was implemented by subtracting the average MFCC3 from MFCC3 during each participant’s utterance. The vowel and digit tasks showed utterance patterns for 3 seconds and the passage task showed utterance patterns for 30 seconds due to the difference in utterance time of each task. As a result, the control group had significantly greater variability in MFCC3 during utterance in all tasks than the MDD group. Conversely, the MDD group showed a monotonous pattern of MFCC3 during utterance in all tasks.

Second, we compared the performance of depression detection for each CNN-based representation model using our data sets. The best performances were obtained with a batch size of 32 in our experiment; thus, all results provided below refer to the models trained with this batch size. Table 6 summarizes the mean and SD of the 10-fold classification performance for each data set and model. The data sets of all tasks showed that the highest performance was observed in the proposed CNN model. In particular, the classification performance of the proposed CNN approach in the passage task showed the best results, with mean accuracy, precision, recall, and *F*_1_-score of 78.14%, 76.83%, 77.90%, and 77.27%, respectively. These results achieved a performance improvement of approximately 8% accuracy compared with the best performance for the conventional ML approach, as reported in Table 5. It also outperformed the other pretrained models.

**Figure 4:**
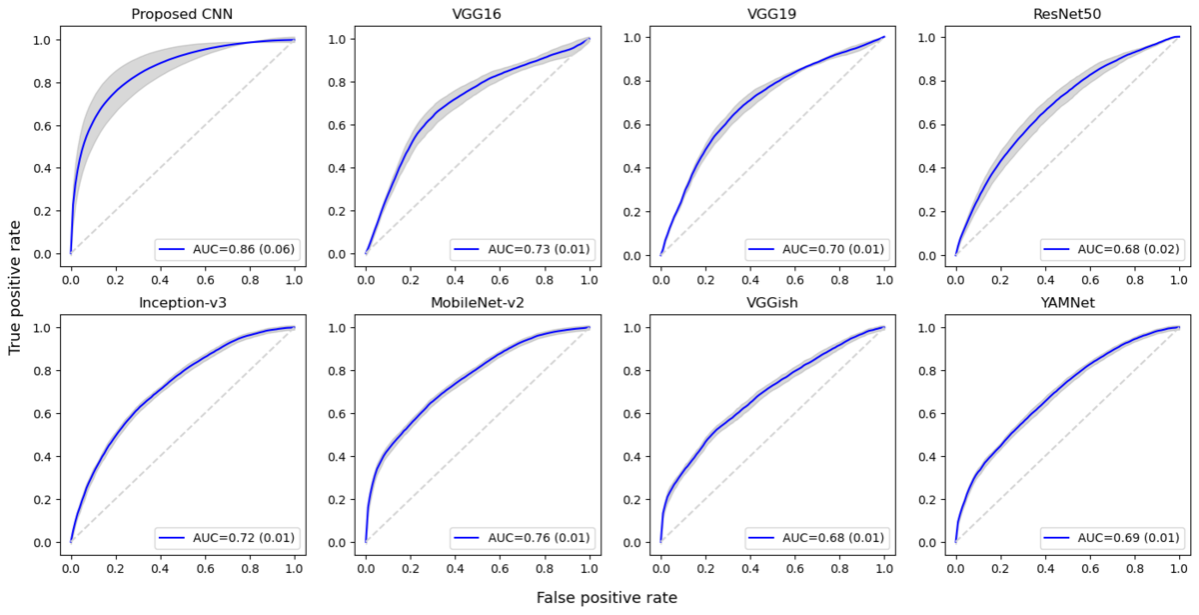
Mean and SD of the receiver operating characteristic curve and area under receiver operating characteristic curve (AUC) based on the convolutional neural network (CNN) models with 10-fold cross-validation in the passage task.

**Figure 5:**
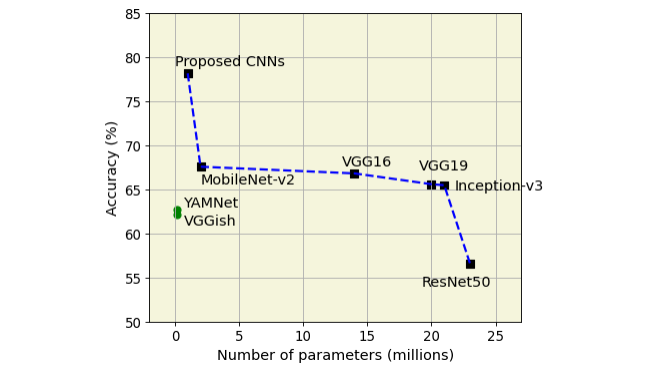
Comparison of the classification accuracy according to the model parameter size in convolutional neural network (CNN) models. ResNet: residual neural network; VGG: visual geometry group; YAMNet: yet another mobile network fully connected.

The receiver operating characteristic curves and AUCs based on the CNN models in the passage task are shown in Figure 4. The mean AUC for the test set of the proposed CNN model was 0.86. These results suggest that the proposed CNN model can capture more information about the depression-specific features contained in text-based speech than the conventional ML approach and the pretrained models. The proposed CNN model achieved a good performance in the passage task; consequently, a more detailed analysis was performed. Figure 5 shows the accuracy based on the model parameter size. In general, it was observed that the accuracy was lower, except for the sound CNN model, when the parameters of the model were larger (YAMNet and VGGish).

In addition, benchmark experiments were performed to compare the performance of depression detection and PHQ-9 score prediction in the benchmark model and the proposed CNN model on audio data sets in a text-dependent setting (read-dependent speech mode) and a text-independent setting (spontaneous mode). A previously reported model [61] that performed well in an independent setting was adopted as the benchmark model. This study provided only the Concordance Correlation Coefficient (CCC) and Root Mean Square Error as an evaluation of the model for predicting the PHQ-9 score. The audio data set from the audio or visual emotion challenge and workshop 2019 detecting depression with artificial intelligence subchallenge, the Extended Distress Analysis Interview Corpus [62], was used as the text-independent setting. The Extended Distress Analysis Interview Corpus contains a multimodal data set of semistructured clinical interviews to evaluate the diagnosis of psychiatric stressors, such as depression, anxiety, and posttraumatic stress disorder.

We first trained both the benchmark and proposed models in a text-dependent setting and then compared the mean performance of 10-folds for depression detection and PHQ-9 score prediction. Here, the performance of depression detection and PHQ-9 score prediction were adopted as the accuracy or *F*_1_-score and the CCC or Root Mean Square Error, respectively [61]. As shown in Table 7, in the case of a text-dependent setting, the classification accuracy of the proposed CNN model was 27% higher than that of the benchmark model. In contrast, the CCC of the benchmark model was higher by 0.25 in the prediction performance of the PHQ-9 score. For the text-independent setting, we conducted an experiment with 163, 56, and 56 training, development, and test participants, respectively, as in the benchmark experiment. The results confirmed that the performance of depression classification and PHQ-9 score prediction in the benchmark model was better than that of the proposed model in a text-independent setting.

These results prove that the model proposed in this study can classify depression better than other models in the dependent setting.

**Table 3:** Comparisons of the performance of machine learning classifiers for Mel-frequency cepstral coefficients (MFCCs), extended Geneva Minimalistic Acoustic Parameter Set (eGeMAPS), and Interspeech Computational Paralinguistics Challenge (COMPARE) feature sets of vowel task (N=318).

Feature and classifier		Test set			
		ACC^a^ (%), mean (SD)	PREC^b^ (%), mean (SD)	REC^c^ (%), mean (SD)	*F*_1_-score^d^ (%), mean (SD)
**MFCCs**					
	SVM^e^	56.56 (3.81)	56.22 (4.01)	55.82 (3.84)	55.43 (3.91)
	LDA^f^	54.69 (2.88)	54.25 (2.99)	53.98 (2.80)	53.65 (2.75)
	kNN^g^	57.19 (5.60)	56.84 (5.85)	56.65 (5.58)	56.47 (5.80)
	RF^h^	60.63 (1.53)	61.24 (2.07)	59.41 (1.44)	58.40 (1.31)
**eGeMAPS**					
	SVM	59.38 (5.93)	59.27 (6.37)	58.59 (6.16)	57.90 (6.58)
	LDA	59.69 (2.60)	59.44 (2.66)	59.16 (2.66)	59.05 (2.71)
	kNN	59.37 (3.13)	59.30 (3.17)	59.29 (3.18)	59.23 (3.18)
	RF	61.25 (2.86)	61.67 (3.20)	60.16 (2.99)	59.32 (3.39)
**COMPARE**					
	SVM	48.75 (3.48)	48.57 (3.62)	48.67 (3.44)	48.26 (3.70)
	LDA	51.25 (3.19)	51.37 (3.46)	51.37 (3.40)	51.07 (3.38)
	kNN	59.38 (5.23)	59.12 (5.42)	58.78 (5.32)	58.61 (5.40)
	RF	72.80 (2.44)	73.70 (2.19)	72.14 (2.64)	72.04 (2.77)

^a^ACC: accuracy.

^b^PREC: precision.

^c^REC: recall.

^d^*F*_1_-score: the weighted average of precision and recall.

^e^SVM: support vector machine.

^f^LDA: linear discriminate analysis.

^g^kNN: k-nearest neighbor.

^h^RF: random forest.

**Table 4:** Comparisons of the performance of machine learning classifiers for Mel-frequency cepstral coefficients (MFCCs), extended Geneva Minimalistic Acoustic Parameter Set (eGeMAPS), and Interspeech Computational Paralinguistics Challenge (COMPARE) feature sets of digit task (N=318).

Feature and classifier			Test set						
			ACC^a^ (%), mean (SD)		PREC^b^ (%), mean (SD)		REC^c^ (%), mean (SD)		*F*_1_-score^d^ (%), mean (SD)
**MFCCs**									
	SVM^e^	53.75 (4.80)		53.82 (4.96)		53.76 (4.87)		53.62 (4.74)	
	LDA^f^	56.25 (5.76)		56.07 (5.84)		55.92 (5.66)		55.87 (5.64)	
	kNN^g^	51.25 (5.80)		50.80 (4.41)		50.82 (4.36)		50.61 (4.44)	
	RF^h^	52.81 (3.26)		51.66 (4.09)		51.63 (3.48)		50.24 (4.24)	
**eGeMAPS**									
	SVM	51.84 (4.62)		51.32 (4.58)		51.30 (4.56)		51.17 (4.60)	
	LDA	48.01 (2.50)		47.95 (2.69)		48.04 (2.64)		47.92 (2.69)	
	kNN	45.36 (3.42)		45.60 (3.48)		45.52 (3.39)		45.13 (3.30)	
	RF	56.21 (3.56)		56.15 (4.89)		55.10 (3.68)		52.96 (4.25)	
**COMPARE**									
	SVM	57.81 (5.09)		57.64 (5.69)		57.08 (5.78)		55.73 (6.77)	
	LDA	62.19 (4.30)		62.32 (4.49)		62.25 (4.41)		62.09 (4.31)	
	kNN	45.31 (4.01)		45.14 (4.03)		45.16 (4.03)		45.10 (4.03)	
	RF	73.44 (1.56)		73.84 (1.79)		72.96 (1.49)		72.99 (1.53)	

^a^ACC: accuracy.

^b^PREC: precision.

^c^REC: recall.

^d^*F*_1_-score: the weighted average of precision and recall.

^e^SVM: support vector machine.

^f^LDA: linear discriminate analysis.

^g^kNN: k-nearest neighbor.

^h^RF: random forest.

**Table 5:** Comparisons of the performance of machine learning classifiers for Mel-frequency cepstral coefficients (MFCCs), extended Geneva Minimalistic Acoustic Parameter Set (eGeMAPS), and Interspeech Computational Paralinguistics Challenge (COMPARE) feature sets of passage task (N=318).

Feature and classifier		Test set			
		ACC^a^ (%), mean (SD)	PREC^b^ (%), mean (SD)	REC^c^ (%), mean (SD)	*F*_1_-score^d^ (%), mean (SD)
**MFCCs**					
	SVM^e^	70.73 (5.93)	70.00 (6.29)	68.18 (6.19)	68.63 (6.63)
	LDA^f^	69.95 (8.38)	70.57 (8.23)	69.79 (8.34)	69.49 (8.60)
	kNN^g^	63.45 (7.76)	63.55 (8.05)	63.25 (7.91)	63.13 (7.87)
	RF^h^	68.54 (7.75)	66.45 (7.40)	67.98 (8.09)	67.27 (8.74)
**eGeMAPS**					
	SVM	59.38 (2.08)	59.35 (2.15)	58.89 (2.15)	58.58 (2.34)
	LDA	58.14 (2.99)	57.96 (3.34)	57.21 (2.96)	56.62 (2.99)
	kNN	61.88 (4.37)	62.11 (4.85)	64.05 (4.71)	60.66 (4.37)
	RF	57.81 (2.21)	57.33 (2.91)	56.74 (2.17)	56.10 (2.36)
**COMPARE**					
	SVM	63.44 (2.44)	63.44 (2.53)	62.96 (2.58)	62.80 (2.71)
	LDA	62.19 (4.93)	62.17 (4.94)	62.10 (4.87)	62.03 (4.90)
	kNN	65.63 (3.13)	65.61 (3.19)	65.49 (3.07)	65.44 (3.06)
	RF	68.75 (1.40)	69.69 (1.27)	67.84 (1.49)	67.60 (1.63)

^a^ACC: accuracy.

^b^PREC: precision.

^c^REC: recall.

^d^*F*_1_-score: the weighted average of precision and recall.

^e^SVM: support vector machine.

^f^LDA: linear discriminate analysis.

^g^kNN: k-nearest neighbor.

^h^RF: random forest.

**Table 6:** Comparisons of the depression detection performance of convolutional neural network (CNN) models in all tasks (N=318).

Data set and models			Test set					
			ACC^a^ (%), mean (SD)		PREC^b^ (%), mean (SD)		REC^c^ (%), mean (SD)	*F*_1_-score^d^ (%), mean (SD)
**Vowel**								
	Proposed CNNs	65.44 (6.58)		64.04 (21.56)		46.66 (24.55)		51.13 (24.38)
	VGG16	47.70 (1.76)		53.16 (1.26)		41.06 (3.83)		46.24 (2.46)
	VGG19	47.97 (0.95)		53.29 (0.83)		43.06 (6.30)		47.37 (4.30)
	ResNet50	47.02 (3.98)		10.99 (21.99)		20.00 (40.00)		14.89 (28.38)
	Inception-v3	56.83 (1.33)		63.13 (1.81)		51.81 (3.01)		56.84 (1.87)
	MobilieNet-v2	57.53 (0.85)		63.34 (0.99)		54.20 (4.66)		58.29 (2.24)
	VGGish	55.93 (0.78)		59.93 (0.77)		60.01 (1.16)		59.99 (0.75)
	YAMNet	62.54 (0.98)		65.78 (0.82)		66.43 (1.96)		66.09 (1.16)
**Digit**								
	Proposed CNNs	66.60 (7.10)		56.79 (28.83)		47.72 (24.99)		51.53 (26.23)
	VGG16	61.69 (1.24)		62.65 (1.19)		48.49 (7.53)		54.32 (5.20)
	VGG19	53.20 (1.62)		60.62 (1.66)		35.94 (6.53)		44.79 (5.90)
	ResNet50	54.15 (9.25)		11.89 (18.16)		30.00 (45.83)		17.03 (26.00)
	Inception-v3	61.69 (1.24)		65.99 (1.50)		58.72 (3.76)		62.06 (2.04)
	MobilieNet-v2	61.92 (2.03)		65.59 (1.64)		60.49 (3.81)		62.9 (2.70)
	VGGish	46.00 (1.74)		49.83 (1.60)		51.26 (2.94)		50.52 (2.14)
	YAMNet	57.37 (0.83)		61.32 (0.73)		55.18 (3.06)		58.04 (1.76)
**Passage**								
	*Proposed CNNs* ^e^	*78.14* (*2.40)*		*76.83* (*3.92)*		*77.90* (*2.73)*		*77.27* (*2.10)*
	VGG16	66.80 (0.40)		65.97 (1.48)		65.48 (3.11)		65.64 (1.05)
	VGG19	65.58 (2.52)		58.14 (2.69)		66.38 (5.16)		61.80 (2.08)
	ResNet50	56.62 (4.84)		79.98 (9.15)		17.11 (18.59)		23.54 (21.24)
	Inception-v3	65.46 (0.28)		63.00 (0.69)		69.86 (2.04)		66.23 (0.60)
	MobilieNet-v2	67.56 (1.28)		65.94 (2.13)		68.98 (3.07)		67.29 (0.97)
	VGGish	62.17 (0.69)		59.96 (0.81)		66.70 (1.70)		63.13 (0.75)
	YAMNet	62.65 (0.52)		60.38 (0.46)		67.39 (1.46)		63.69 (0.74)

^a^ACC: accuracy.

^b^PREC: precision.

^c^REC: recall.

^d^*F*_1_-score: the weighted average of precision and recall.

^e^The accuracy, precision, recall, and *F*_1_-score of the model that the best performance are italicized for emphasis.

**Table 7:** Performance comparisons of a bench model [61] and a proposed convolutional neural network (CNN) model for depression detection and Patient Health Questionnaire-9 score prediction on audio data sets in a text-dependent setting (read-dependent speech mode) and a text-independent setting (spontaneous mode; N=318).

Models	Data sets								
	Text-dependent setting (mean of 10-fold)					Text-independent setting (single fold)			
	ACC^a^ (%)	*F*_1_-score^b^ (%)	CCC^c^	RMSE^d^	ACC (%)		*F*_1_-score (%)	CCC	RMSE
Proposed CNNs model	78.14	77.27	0.28	9.21	56.82		37.84	0.287	5.53
GCNN-LSTM^e^ [61]	51.65	50.90	0.43	8.10	58.57		39.78	0.497	5.70

^a^ACC: accuracy.

^b^*F*_1_-score: the weighted average of precision and recall.

^c^CCC: Concordance Correlation Coefficient.

^d^RMSE: Root Mean Square Error.

^e^GCNN-LSTM: Gated Convolutional Neural Network-Long Short Term Memory.

## Discussion

### Principal Findings

Our results suggest that the speech characteristics obtained through text-dependent speech can be a promising biomarker for MDD. In this study, we recorded speech data using a mobile phone with predefined text-based reading speech tasks and confirmed the potential for automatically detecting depression based on the recorded data and a deep learning approach. As previous studies have suggested the possibility of detecting depression in speech data [21,25,27,30,57,63], we could easily access speech data for depression detection. We extended the research from image-oriented studies to speech studies using 2D CNN architectures. The sound signal was then transformed into an LM spectrogram as a common denominator in an image application and was used as the input to a 2D CNN.

In the passage task, the approach that adopted the CNN model improved performance by approximately ≥7% compared with the conventional ML approach. Compared with conventional ML methods for evaluating handcraft-based extracted feature sets, the acoustic feature extraction approach of CNNs generally exhibits features in a broader spectrum and shows higher performance, as shown in terms of classification accuracy. When the performances of the 3 text-dependent reading tasks were compared (Table 6), the CNN model also showed higher classification performance in the audio sample reading the passage task. The passage task is a neutral expression standardized with Korean phrases that contain balanced proportions of consonants and vowels, and because the text is longer than the vowel and digit task, it can be used as a more reliable depression diagnosis protocol. These results provide opportunities for performance improvement if further development and optimization of CNN architecture with more spectrum of text-dependent speech data sets are implemented in audio-based depression detection studies.

The features of the audio samples were extracted from well-known pretrained models (VGG16, VGG19, Inception-v3, ResNet50, MobileNet-v2, VGGish, and YAMNet), such as features extracted through a customized CNN model, and the classification performance was compared. We found that the feature set extracted through pretrained models from all data sets of text-based speech tasks did not significantly affect the classification performance, as shown in Table 6. The shallow network we built showed good classification performance in detecting depression, with fewer resources in the text-based speech tasks. These results show that the available CNN models designed and trained on the ImageNet data set are not necessarily critical for good performance in tasks based on nonimage data, such as a spectrogram. ResNet50 did not perform well in the classification of all 3 tasks. In general, this can be justified by the need for a larger data set to compute the weights, as shown in Figure 5.

Audio samples in the passage task achieved 78% accuracy in depression detection (Table 6), and we observed that the CNN model proposed in the text-dependent setting was advantageous in detecting depression based on the benchmark experiments and comparisons (Table 7). However, our model could not be generalized for the prediction of PHQ-9 score and in spontaneous situations. Text-dependent read speech is more private and can be easily obtained with a smartphone compared with voluntary speech containing personal information [30,62]. Implementing these text-dependent speech tasks has the advantage of reducing acoustic variability and enabling more precise analysis because they acquire speech in a controlled manner and can standardize speech acquisition to produce consistent results in detecting depression. We presented the possibility of detecting depression by recording a text-reading-based audio sample from a smartphone. If this approach is applied to daily life, anyone can be screened for depression anywhere without difficulty.

Previous studies have focused on developing depression detection models based on open cohort data [7,21]. However, because these data sets collected speech data from English-speaking countries, their application to non-English-speaking countries may be difficult. Owing to the linguistic or cultural differences between English-speaking and non-English-speaking countries, it is necessary to collect a corpus suitable for non-English-speaking countries and analyze depression detection. Thus, we collected a Korean-based audio corpus. To the best of our knowledge, there are few cases of speech corpus for detecting depression in Korea, and our data set may serve as the starting point for improving access to psychiatric treatment in Korea.

### Limitations

Our study had some limitations. First, we used a small number of voice samples in the experiments; thus, we are currently recruiting additional participants to collect more data to run deep learning. With further research in this cohort, we plan to report the outcomes of developing ML techniques for disease diagnosis and severity prediction. Second, all the audio samples were recorded in a quiet environment. Extended studies are needed to apply this approach to other recording environments (eg, in the wild and noisy conditions). In addition, we plan to explore different strategies for combining our speech-based systems with various information, such as video or physiological signals. Approaching the multimodal detection will present a robust framework that operates under more precise and natural conditions. We believe that these efforts will help to build more robust predictors of MDD for daily life in the future.

### Conclusions

This study has opened new opportunities to identify speech markers related to the assessment of depression through readily obtainable speech patterns. This study also presented an approach to automatically detect whether a person has depression by analyzing their speech. We acquired audio samples from 318 participants with depression and healthy controls based on the Korean text-dependent read speech tasks using a smartphone and analyzed their association with depression. We found that there are many benefits of learning audio acoustic patterns and detecting depression using deep learning. This approach has the potential to reduce depression and shows that it is powerful and effective in ADD via speech.

## Abbreviations

ADD: automatic depression detection
AUC: area under receiver operating characteristic curve
CCC: Concordance Correlation Coefficient
CNN: convolutional neural network
COMPARE: Interspeech Computational Paralinguistics Challenge
eGeMAPS: extended Geneva Minimalistic Acoustic Parameter Set
HAM-D: Hamilton Depression Rating Scale
kNN: k-nearest neighbor
LDA: linear discriminate analysis
LM: log-Mel
MDD: major depressive disorder
MFCC: Mel-frequency cepstral coefficient
ML: machine learning
PCM: Pulse-Code Modulation
PHQ-9: Patient Health Questionnaire-9
RF: random forest
SVM: support vector machine
